# Physiopathological approach to infective endocarditis in chronic hemodialysis patients: left heart versus right heart involvement

**DOI:** 10.1080/0886022X.2017.1305410

**Published:** 2017-03-24

**Authors:** Yassamine Bentata

**Affiliations:** Department of Nephrology, Medical School, University Mohammed the First, Oujda, Morocco

**Keywords:** Infective endocarditis, chronic hemodialysis, left heart, right heart, physiopathology

## Abstract

Infectious endocarditis (IE), a complication that is both cardiac and infectious, occurs frequently and is associated with a heavy burden of morbidity and mortality in chronic hemodialysis patients (CHD). About 2–6% of chronic hemodialysis patients develop IE and the incidence is 50–60 times higher among CHD patients than in the general population. The left heart is the most frequent location of IE in CHD and the different published series report a prevalence of left valve involvement varying from 80% to 100%. Valvular and perivalvular abnormalities, alteration of the immune system, and bacteremia associated with repeated manipulation of the vascular access, particularly central venous catheters, comprise the main factors explaining the left heart IE in CHD patients. While left-sided IE develops in altered valves in a high-pressure system, right-sided IE on the contrary, generally develops in healthy valves in a low-pressure system. Right-sided IE is rare, with its incidence varying from 0% to 26% depending on the study, and the tricuspid valve is the main location. Might the massive influx of pathogenic and virulent germs via the central venous catheter to the right heart, with the tricuspid being the first contact valve, have a role in the physiopathology of IE in CHD, thus facilitating bacterial adhesion? While the physiopathology of left-sided IE entails multiple and convincing mechanisms, it is not the case for right-sided IE, for which the physiopathological mechanism is only partially understood and remains shrouded in mystery.

## Introduction

Brescia M. J. described the first case of infective endocarditis in a chronic hemodialysis patient in 1966, although dialysis had started a few years earlier.^1^ In this case, IE occurred in a patient who was 28 years old, had 10 months of duration of dialysis and a native arteriovenous fistula. IE was diagnosed with rheumatic aortitis and mitral valvulitis and the patient died.

For several decades, it has been well established that cardiovascular diseases and infections are the two main causes of mortality in chronic hemodialysis (CHD).^2^ Up to 40–50% of patients with end-stage renal disease (ESRD) die of cardiovascular disease (CVD) and the mortality rate due to CVD in patients on hemodialysis is 10–30 times higher than that in the general population.^3^ Infective endocarditis (IE), a complication that is both cardiac and infectious, occurs frequently and is associated with a heavy burden of morbidity and mortality in CHD. Patients receiving long-term hemodialysis (HD) are at increased risk for IE with an age-adjusted incidence ratio of 17.9 compared with the general population.^4^ About 2–6% of chronic hemodialysis patients develop IE and the incidence is 50–60 times higher among CHD patients than in the general population.^5^ Despite improvements in the prognosis of IE in the general population, the mortality rate is still much higher in hemodialysis populations due to a combination of factors, including extensive co-morbidities and virulence of germs; the mortality rate reported in different series published is 25–77%.^6–8^

The patients’ immunodeficiency, repeated manipulation of the vascular access, type of vascular access used, and pre-existing valvular changes combine to place CHD patients at high risk for IE.^9–11^ On first thought, one might expect IE in CHD to occur in the right heart because of the frequent manipulation of the central venous catheter, similar to what occurs in intravenous drug use by drug addicts. In reality, however, the IE in CHD affects the left heart in more than 90% of cases.^12–14^

Despite the high frequency and severity of IE in CHD, few series have been published in the literature over the last 30 years. Does IE remain under diagnosed or easily diagnosed in this context? Does it not raise enough interest among practitioners? Understanding of the pathophysiology of IE in HDC is not complete and several points remain unclear and unanswered. In addition, none of the published series distinguishes among the clinical, biological, and bacteriological characteristics of left heart IE and those of right heart IE, thus treating them as if they were part of the same physiopathological entity. However, we think that there are indeed two distinct entities and as in the general population, these two entities are distinct in all physiopathological, therapeutic and prognostic terms. Consequently, this physiopathological approach to IE in CHD will be presented according to the site of cardiac involvement by differentiating between left-sided IE and right-sided IE, since these two forms of IE arise from different physiopathological mechanisms. Throughout this review, we shall present, understand, and discuss the pathophysiological mechanisms involved and the distinctive elements between IE of the left heart and the IE of the right heart.

## Left-sided infective endocarditis (LSIE) in chronic hemodialysis

### Epidemiology

The left heart is the most frequent location of IE in CHD, and the different published series report a prevalence of left valve involvement varying from 80% to 100%.^12–15^ The mitral valve is the main site of IE in CHD and approximately 50% of LSIE affects the mitral valve followed by involvement of the aortic valve, while the concomitant involvement of the mitral and aortic valves has been found in 10–25% of cases.^16–20^Table 1 shows the valvular sites of IE in CHD as reported by the main studies published over the past 50 years that include more than five cases. The most important series published in the literature that specified the location of endocarditis is that of Kalakannan who reported a series of 69 cases of IE in patients on chronic hemodialysis.^8^

**Table 1.** Main characteristics and location of IE in chronic hemodialysis patients based on the presence of valvular vegetations detected by transthoracic echocardiography and/or transesophageal echocardiogram.

Series	Period of study (years)	Number of cases (*N*)	Mean age, years	Gender, male (%)	Diabetes (%)	IDU (%)	Duration of dialysis, months	Cardiac valvular disease (%)	Mitral valve (%)	Aortic valve (%)	Mitral and aortic valves (%)	Tricuspid valve (%)	MSSA MRSA SE (%)	No native vascular access (%)	Overall IH mortality or at 30 d (%)
Leonard et al. (USA)^7^	1966–1972	9	55	67	NR	NR	28.6	22.3	11.1	33.3	33.3	22.3	55.5	88.8	77.7
Doulton et al. (UK)^12^	1980–2002	30	54.1	60.7	28.5	NR	46.3	NR	43.3	36.6	16.1	0	63.3	52.1	30
Mc Carthy et al. (USA)^61^	1983–1997	20	63	76.4	35.2	NR	24	90	45	25	20	10	47	88.2	47
Takahashi et al. (Portugal)^73^	1985–1989	19	–	–	–	NR	19.5	NR	42.1	31.5	–	–	75	60	68.4
Chang et al. (Taiwan)^20^	1988–2002	20	64	65	45	NR	13	NR	55	15	10	20	60	75	60
Hanslik et al. (France)^13^	1988–1994	30	62	NR	NR	NR	NR	NR	43	40	17	0	53	NR	43
Robinson et al. (USA)^19^	1990–1997	20	55	30	45	5	3.4^a^	NR	50	30	15	25	55	95	30
Maraj et al. (USA)^6^	1990–2000	32	54	44	NR	16	7.6^a^	NR	53	38	15.3	13	80	97	25
Baroudi et al. (USA)^69^	1990–2006	59	57.3	47	59	7	52.9	NR	63	17	13.6	5.1	45.7	91.6	37.9
Kamalakannan et al. (USA)^8^	1990–2004	69	56	45	37.7	11.6	37 10^a^	NR	49.3	21.7	13	10.1	72.3	88.4	49.3
Tao et al. (China)^74^	1990–2009	6	52	66.6	0	NR	27^a^	20	50	33.4	16.6	0	33.3	83.4	33.3
Spies et al. (Hawaii)^18^	1991–2001	40	59	35	50	0	39 22^a^	38	52	23	20	5	65	48	52
Fernandez et al. (Uruguay)^21^	1995–2000	21	61	47.6	9.5	0	56	NR	43	28.5	9.5	19	67.5	77	28.6
Rekik et al. (Tunis)^14^	1997–2006	16	52	62.5	6.2	6.2	27.3	25	56.2	25	12.5	6.2	70.2	25	43.7
Jones et al. (UK)^22^	1998–2011	40	55	52.2	33.3	NR	57.4	33.3	30.9	42.8	NR	9.5	57.1	65	29.2
Nori et al. (USA)^15^	1999–2004	54	60	52	42	12	6^a^	NR	50	43	9.2	19	40	96	36.5
Oun et al. (UK)^16^	2000–2013	29	57	59	24	NR	30	79.3	48.3	17.2	20.7	13.8	75.9	69	37.9
Mangoni et al. (Italy)^55^	2004–2011	42	66	69	42.9	11.9	NR	NR	35	35	NR	15	67.6	31	26.2
Kremery et al. (Slovakia)^17^	23 years	28	NR	NR	3.6	0	NR	NR	50	50	NR	92.9	17.9	NR	32.1
Bentata et al. (Morocco)^62^	2010–2016	9	38	77.7	33.3	0	18	NR	22.2	22.2	0	55.6	22.2	77.7	55.5

IDU: intravenous drug users; Cardiac valvular disease was defined by existing of valvular calcification, rheumatic disease, valve prosthesis; MSSA: methicillin-resistant *Staphylococcus aureus*; MRSA: methicillin-resistant *Staphylococcus Aureus*; SE: *Staphylococcus epidermidis*; no native vascular access included temporary catheter, tunneled catheter and/vascular graft; NR: not reported; IH: in-hospital.

^a^Duration of vascular access use.

### Pathogenesis

The rarity of endocarditis, despite the frequency of transient bacteremia, indicates that the intact valvular endothelium is resistant to colonization by microorganisms. Lesion development in the valvular endothelium thus seems to be an essential factor in development of IE. We shall present the different factors that cause damage to the valvular endothelium and foster bacterial growth in CHD.

Susceptibility to endocarditis in patients undergoing hemodialysis is multifactorial, with several factors playing an important role in the predisposition and the development of IE. Some of these factors are related to the quality of heart valves and others to the nature of the vascular access for hemodialysis and the state of fragility of the patient.

IE occurs in more than 90% of cases in a native valve, a valve that although certainly native, is the site of many alterations, and a degenerative process, which are both hemodynamic and lesional.^21^^,^^22^ Valvular calcifications play an essential role in the pathogenesis of LSIE, representing one of the most important parameters of chronic kidney disease. Mitral annular calcification (MAC) and aortic valve calcification (AVC) are the main valvular calcifications observed in ESRD.^23–26^ In patients undergoing long-term HD therapy, valve calcification is markedly more common, with reported prevalence rates of 50–80%, which are strongly associated with mortality.^27–29^ In some studies, the prevalence is even higher; Kraus reported a prevalence of mild/moderate mitral valve and aortic valve calcification in 275 patients with ESRD on hemodialysis of 98.8% and 94.4%, respectively.^30^

These valvular and perivalvular abnormalities are closely linked to alterations of phosphocalcic metabolism, atherosclerosis, and inflammation. In fact, chronic kidney disease before the onset of ESRD leads to major phosphocalcic disturbances such as hypocalcemia, hyperphosphatemia, elevated calcium-phosphorus product, elevated parathormone, and elevated Fibroblast growth factor 23. All these abnormalities are related largely to reabsorption failure, renal excretion of calcium and phosphorous, and a faulty renal production of calcitriol.^31^^,^^32^ Recently, valvular calcification has also been associated with abnormalities of various calcium regulatory factors such as the level of Fetuin-A. Fetuin-A is a serum protein that stabilizes calcium–phosphate in a complex, which enables its clearing by the phagocytic system and its values decreased in patients with ESRD.^33^^,^^34^

Cardiac valve calcification, frequently seen in patients undergoing dialysis, has also been regarded as a marker of atherosclerosis because the calcification process is generally similar to the process leading to atherosclerotic plaque calcification and it is characterized by the presence of monocytes, macrophages, and osteoblast-like cells.^27^^,^^35^ Recent studies also suggest an important role for inflammation and malnutrition in the development of valvular calcifications.^28^^,^^33^^,^^36^ Other factors contribute to the development of these valvular and perivalvular abnormalities such as advanced age, male gender, ethnicity, duration of hemodialysis, smoking, diabetes, hypercholesterolemia, iron overload, peripheral vascular disease, cardiac dysrhythmia, and arterial hypertension.^30^^,^^37–40^

Increased stress on the mitral and aortic valves and perivalvular structures accelerates the lesion production and degenerative processes.^41^ The increase of this valvular and perivalvular stress is associated with an increase in left ventricular pressure, itself secondary to systemic hypertension, cardiomyopathy, aortic stenosis, and fluid overload. All these situations are frequently observed in patients with ESRD and/or CHD contributing to the development and progression of valvular calcification.

All these valvular and perivalvular abnormalities together constitute a strong predisposing factor for IE and the valvular endothelium thus becomes sensitive to bacterial colonization, but these conditions are not sufficient to engender IE and other factors must also intervene. Alteration of the immune system and bacteremia associated with repeated manipulation of the vascular access, particularly central venous catheters in CHD patients, comprise the two main factors. Indeed, immune dysfunction in uremia is a state of both immune activation and immune suppression, an imbalance that leads to increased susceptibility to infections, as well as to inflammatory responses, which, when systemic in nature, are closely related to cardiovascular disease.^42^^,^^43^

More than half of IE cases in CHD are preceded by an episode of bacteremia that originate in more than 70% of cases in a central venous catheter for hemodialysis. In CHD, catheter use still remains very high and international recommendations that advocate catheter use in less than 10% of patients in a hemodialysis center are difficult to achieve.^44^ Dialysis-related bloodstream infections account for up to 75% of deaths caused by infections among patients on HD.^45^

There are four types of accesses for hemodialysis: temporary dialysis catheter, tunneled (permanent) dialysis catheter, arteriovenous fistula, and arteriovenous graft. It is established that temporary HD vascular catheters are associated with the highest rate of access-related bacteremia, followed by permanent central venous catheters, synthetic grafts, and native fistula.^46–48^ Catheter-related bacteremia (CRB) is the most common and, indeed, the most dreaded of complications. The incidence of CRB has been reported by various studies at 2.5–5.5 episodes/1000 catheter days or 0.9–2.0 episodes/person/year. This risk is two to three-fold higher for temporary HD catheters.^49^^,^^50^ There are multiple risk factors for CRB, including previous episodes of bacteremia, older age, diabetes, malnutrition, iron overload, longer duration of catheter use, and peripheral atherosclerosis.^51^ Repeated manipulation of the vascular access in CHD, especially when aseptic measures are not applied, is a major risk factor for CRB.^52^ CRB is one of the strongest parameters in the development of IE and when we reduce the frequency of CRB we can thereby drastically reduce the occurrence of IE.

It is important to note that despite the great variability between countries concerning the bacteriological epidemiology, *Staphylococcus aureus* remains the main cause of both vascular access-related bacteremia among patients receiving long-term HD and of IE, which complicates staphylococcal bacteremia in 5–10% of cases.^52^^,^^53^ The other germs incriminated are coagulase negative Staphylococcus, Streptococcus, Enterococcus, *Klebsiella pneumonia,* and *Pseudomonas aeruginosa.*^54^ The appearance of methicillin-resistant *S. aureus* is continually increasing and varies according to the series from 5% to 40%. Involvement of Candida and other species is infrequent and unusual. However, the incidence of negative blood cultures due to previous antibiotic therapy for CRB that preceded IE, or for infection at a different site remains relatively high and complicates the therapeutic management of endocarditis.

### Risk factors

The advanced age frequently encountered in the IE of the general population is not found in the dialysis population. On the contrary, the average age is relatively young in chronic hemodialysis patients. But probably, the average age varies according to the epidemiology of the ESRD of each country and thus explains this observed difference (38 years in morocco, 52 years in Tunisia, and 63 years in United States of America).

Diabetes is one of the most frequently associated risk factors and its prevalence is higher in chronic hemodialysis patients with IE and varies from 3% to 50%.^8^^,^^15^^,^^17^^,^^55^ Certainly, diabetic immunodepression is added to the sphere of hemodialysis immunodepression to promote IE and aggravate the prognosis. All co-morbidities and circumstances leading to immunodepression in chronic hemodialysis patients can contribute to the occurrence of endocarditis, such as viral hepatitis B and C, drug use, neoplasia, and bacteremia, even if the initial infectious point is not the central venous catheter.

Inadequate dialysis, particularly in developing countries where access to three sessions a week is sometimes difficult, can contribute to worsening patient immunity.

## Right-sided infective endocarditis (RSIE) in chronic hemodialysis

### Epidemiology

While the physiopathology of left-sided IE entails multiple and convincing mechanisms, it is not the case for right-sided IE, for which the physiopathologic mechanism is only partially understood and remains shrouded in mystery.

IE of the right heart represents 5–10% of IE in the general population. About 90% of RSIE occurred in subjects who were injecting drug users (IDUs) and 10% of RSIE occurred in patients with intravascular catheter for hemodialysis, implanted ports for chemotherapy, vascular prostheses, or intracardiac devices.^56–58^

In the series of Jiang et al. in China, of 412 patients with IE who were candidates for surgical treatment in a period of 10 years, 35 (8.5%) had a right-sided IE, including one patient on hemodialysis, that is 2.8%.^58^ In the series of Musci et al. in Germany, in a 20-year period, 57 patients had an isolated IE of the right heart, including three on hemodialysis, that is 5.2%.^56^ In the series of Dawood et al. in the USA, in a 10-year period, 56 patients had IE (among 322 with IE) including two on CHD, that is 3.6%.^59^

In CHD, RSIE is rare, with its incidence varying generally from 0% to 26% depending on the study, and the tricuspid valve is the main location.^12^^,^^19^^,^^20^^,^^60^^,^^61^ Some studies however, have reported a very high incidence of more than 50%.^17^^,^^62^

### Pathogenesis

While LSIE develops on altered valves in a high-pressure system, RSIE on the contrary, generally develops in healthy valves that are in a low-pressure system. In CHD, the right valves are not changed and valvular and perivalvular abnormalities are absent. What then, are the physiopathological mechanisms explaining IE of the right heart in CHD? RSIE, aside from that occurring on intracardiac devices or among IDUs remains a rare and poorly understood entity. Conventional belief is that in IDUs the tricuspid valve is the first valve encountered and damaged after the intravenous injection of particulate material.^63^ In addition, endothelial damage may result from so-called jet lesions due to turbulent blood flow or may be provoked by electrodes, intracardiac devices or catheters, or by repeated intravenous injections of solid particles in IDUs.^64^

Although these hypotheses adequately explain the pathogenesis of RSIE in IDUs, they are insufficient to explain IE in CHD because the conditions are identical to those underlying left-sided IE in CHD: inflammation, atherosclerosis, altered left valves, repeated manipulation of a hemodialysis central venous catheter, with the pathogenic germ often being a Staphylococcus. Is it related to an individual predisposition involving the expression of cytokines, the formation of immune complexes, cellular dysfunction and sensitivity to the pathogen?^65–67^ Might the tricuspid valve be altered in certain CHD patients? Vegetations occur more often in right-sided IE and are often large as the low pressure prevailing in the right ventricle encourages the increase in vegetation size.^55^^,^^57^^,^^68^ The prevalence of IDUs in CHD varies from 0% to 16% and the studies that reported high incidence of RSIE had a high frequency of IDUs.^6^^,^^8^^,^^15^^,^^55^^,^^69^ Nori et al. reported in their series of 54 cases of IE in CHD, 19% of RSIE and 12% of IDUs.^15^ Injectable drugs probably induce valvular endothelial lesions that favor bacterial overgrowth in staphylococcal septicemia.

Pulmonary hypertension (PHT) is another risk factor that probably plays an important role in pathogenesis of right-sided IE and its prevalence ranges from 18.8% to 68.8% in CHD.^70^ The three major causes of PHT are increased cardiac output related to hypervolemia and arteriovenous fistula, increased pulmonary vascular resistance mainly related to uremic endothelial dysfunction, pulmonary artery calcifications, and elevated pulmonary capillary wedge pressure caused by heart failure or mitral valve disease.^71^ This PHT may cause hemodynamic changes and blood flow turbulence in the right heart that lead to the vulnerability of right cardiac valves and then the occurrence of IE.

Although the microbiologic profile of RSIE does not differ from that of LSIE, *S. aureus* remains the major microbiological cause of RSIE in HDC patients.^72^ Might the massive influx of pathogenic and virulent germs via the central veins to the right heart with the tricuspid being the first contact valve, have a role in the physiopathology of IE in CHD, thus facilitating bacterial adhesion?

Some studies, however, do not report any case of RSIE even for large series.^73–75^ Aboot et al. studied risk factors of IE in a large cohort of CHD patients (*n* = 2075) but without specifying the site of IE. They found that a history of congestive heart failure, dysrhythmia, and decreased serum albumin at initiation of dialysis were independent factors associated with hospitalization for bacterial endocarditis.^4^ In this study, the risk for IE was constant over time after the initiation of dialysis. Table 2 summarizes the main physiopathological and risk factors that contribute to development of IE in chronic hemodialysis patients.

**Table 2:** The main physiopathological and risk factors of infective endocarditis in chronic hemodialysis patients.

Physiopathological and risk factors
Factors related to underlying valvular disease
Valve calcification
Rheumatic valve
Prosthetic valve
Other valvular diseases (insufficiency, stenosis, etc.)
Factors related to vascular access
Temporary catheter
Tunneled catheter
Vascular graft
Staphylococcus bacteremia related to vascular access
Long duration of hemodialysis
Advanced age
Male gender
Ethnicity (White)
Diabetes mellitus
Anemia
Inflammation
Atherosclerosis
Malnutrition
Iron overload
Low-serum albumin
Peripheral vascular disease
Dysrhythmia
Intravenous drug users

None of the published studies on chronic hemodialysis patients have ever separately studied the clinical, biological, and microbiological aspects of RSIE and LSIE. Consequently, it is very difficult for us to affirm the physiopathological factors in RSIE during CHD, for which the prevalence is considerably lower than for IE of the left heart.

It is important to highlight that the diagnosis of IE requires a high index of suspicion and is made using the redefined Dukes criteria combined with echocardiographic imaging, which itself fails to distinguish between RSIE and LSIE and views both as the same entity.^76^

## Conclusion

The physiopathology of infective endocarditis remains largely unclear. The physiopathological mechanisms proposed until now do not completely explain IE and even less the reasons for which certain CHD patients develop IE of the right heart and others of the left heart, but bacteremia related to catheter infection remain the major risk factor for developing IE in CHD. What is certain is that one must not miss the diagnosis of IE and that it must be considered in any CHD patient who presents with fever, pulmonary symptomatology, anemia, or other suggestive clinical and biological signs and also during an episode of staphylococcal septicemia. Thus, patients receiving hemodialysis should be at the forefront of preventive strategies to reduce health-care associated bacteremia, and high clinical suspicion for infective endocarditis is warranted in this group.
